# Exploring the Role of Sodium Dodecyl Sulfate Surfactant in Enhancing the Fluorescence Properties of Besifloxacin Fluoroquinolone; Application to Eye Formulations and Artificial Aqueous Humor

**DOI:** 10.1007/s10895-025-04310-1

**Published:** 2025-05-09

**Authors:** Islam M. Mostafa, Demiana W. Fakhry, Mohamed A. Abdelshakour, Deena A. M. Nour El-Deen

**Affiliations:** 1 Analytical Chemistry Department, Faculty of Pharmacy, Minia University, Minia, 61511 Egypt; 2 Analytical Chemistry Department, Faculty of Pharmacy, Minia National University, New Minia, 61511 Egypt; 3 Department of Pharmaceutical Analytical Chemistry, Faculty of Pharmacy, Sohag University, Sohag, 82524 Egypt

**Keywords:** Besifloxacin, Micellar system, Ophthalmic solution, Aqueous humor

## Abstract

An ultrasensitive, selective, rapid, and cost-effective spectrofluorimetric approach is established and validated to quantify the ophthalmic fluoroquinolone antibiotic, besifloxacin (BN), in its ophthalmic preparations and artificial aqueous humor. Unlike previously reported methods that require costly fluorogenic derivatization reagents, lengthy analytical procedures, or expensive instrumentation, the proposed approach offers a simple and highly sensitive alternative method for detecting BN. The suggested method relies on using 2.0% w/v sodium dodecyl sulfate (SDS) as a micellar system in an acetate buffer solution (pH 3.6) to boost the fluorescence intensity of BN at λ_em_ of 446 nm. The fluorescence intensity of BN was remarkably amplified by around 2.6 folds compared to the BN in acetate buffer without SDS surfactant. The linearity range of the developed method was 5.0–100 ng/mL with detection and quantitation limits of 0.64 and 1.93 ng/mL, respectively. Moreover, the proposed micellar system has been effectively used for the assay of the BN in eye drops and artificial aqueous humor and the achieved percentage recoveries for artificial aqueous humor samples were in the range of 98.82 ± 1.89–103.08 ± 1.60%.

**Supplementary Information:**

The online version contains supplementary material available at 10.1007/s10895-025-04310-1.

## Introduction

Besifloxacin (BN) is a broad-spectrum fluoroquinolone antibiotic primarily used for the treatment of bacterial conjunctivitis (commonly known as pink eye). It is formulated as an ophthalmic suspension for topical application, ensuring localized drug delivery while minimizing systemic absorption and resistance development [1–3]. It has significant action against numerous gram-positive, gram-negative, and anaerobic organisms, such as *methicillin-resistant Staphylococcus aureus and Staphylococcus epidermidis*, along with a limited propensity for bacterial resistance [1–3]. After topical application, BN reaches high, persistent concentrations in the tear fluid and conjunctiva with minimal systemic exposure. It is given three times a day for five days to treat acute bacterial conjunctivitis in both adults and children [1, 4–7].

Most of the reported detection methods for BN are chromatographic methods that involve expensive chromatographic reagents with high purity for elution and need skilled workers [8–13].

Additionally, various spectroscopic approaches have been introduced to estimate the amount of BN in bulk and various pharmaceutical formulations including spectrophotometric [14, 15] and fluorimetric [16, 17] approaches. However, spectrophotometric methods suffer from low sensitivity, and previous spectrofluorimetric techniques for BN detection often relied on derivatization reactions that consume time or were based on fluorescence quenching mechanisms, which affect the selectivity and sensitivity. Furthermore, cyclic and differential pulse voltammetric electrochemical tools were employed for detecting BN using a gold nanoparticle-modified carbon paste electrode [18]. However, these methods are less feasible for routine pharmaceutical analysis since they frequently use costly nanomaterials and intricate electrode modifications.

Micelle production is a key approach for fluorimetric investigation of medicines and chemicals [19–23]. Micelles, made up of surfactant molecules, produce a distinct microenvironment that greatly affects the fluorescence properties of specific chemicals [24–26]. This method uses micelles to modify the solubility, chemical structure, and fluorescence emission of several compounds, improving sensitivity and selectivity in detection. Micelle production has enabled researchers to identify and measure medicines, contaminants, heavy metals, and biomolecules [27]. The interaction between these chemicals and micelles alters their fluorescence behavior, enabling sensitive and exact investigation. The micelle-based spectrofluorimetric methods have been extensively used in pharmaceuticals, environmental monitoring, and biochemical research to identify tiny levels of compounds [28, 29].

In contrast to the limitations of the reported method for detecting BN and based on the above-mentioned merits of micelle impact on the fluorescence behavior of different ‎substances. This method leverages the fluorescence-enhancing effect of micelles on the BN fluorescence behavior without requiring expensive fluorogenic derivatization, complex sample preparation, or sophisticated instrumentation. Herein, we investigated the effect of SDS surfactant on the fluorescence behavior of ‎BN to develop a simple, sensitive spectrofluorimetric method for determining BN in ophthalmic ‎preparations and spiked aqueous humor.

## Experimental

### Apparatus

A Jasco FP-8350 spectrofluorometer was used to take the spectrofluorimetric measurements. Both the emission and excitation monochromators had a 10 nm width. A Jenway 3510 pH meter (Staffordshire, UK) was used to control the pH. Weighing was done using a Mettler Toledo 5-digit balance (Greifensee, Switzerland).

### Chemicals and Reagents

Eva Pharma Company presented BN (purity 99.50%‎) as a gift (Cairo, Egypt). The dosage form, Pixivance^®^ eye drops with batch no. 2,404,169, which was marked as containing 6.0 mg/mL (5.0 mL), was bought from the local market. Sodium dodecyl sulfate (SDS) El-Nasr Chemical Co. (Cairo, Egypt) was prepared as a 2.0% w/v aqueous solution. 0.2 mol/L sodium acetate solution and 0.2 mol/L acetic acid solution were combined to create buffer solutions with the necessary pH. Cetyl trimethyl ammonium bromide (CTAB, 99.0%), Tween 80 and β-cyclodextrin (β-CD) were purchased from Winlan El-Nasr Chemical Co. (Cairo, Egypt). Methanol and ethanol were obtained from Sigma Aldrich (Germany). Acetonitrile and acetone were obtained from El-Nasr Chemical Co. (Cairo, Egypt).

### Standard Solutions Preparations

In a 100 mL volumetric flask, a stock solution (0.1 mg/mL) of BN was prepared by dissolving 10.0 mg of the pure drug in 100 mL of distilled water. Further dilutions were done with the same solvent to create working solutions within the concentration range of 50-1000 ng/mL.

### General Analytical Procedure

Aliquot volumes of the working solutions were transferred into a series of 10 mL volumetric flasks, and then 1.0 mL of 0.2 mol/L acetate buffer ‎‎(pH 3.6) and 1.5 mL of SDS solution were added to each flask. The solutions were mixed well, and thus, 5.0–100 ng/mL was the final concentration range. The fluorescence intensity was measured at 446 nm after excitation at 274 nm.

### Assay of BN in Eye Drop Preparations

An aliquot volume (1.7 mL) of Pixivance^®^ eye drops and 60 mL of distilled water were added to a 100 mL calibrated flask, and the mixture was then well combined. The solution was filtered numerous times after being adjusted with distilled water to the appropriate level. To get the BN concentrations within the range of the technique, the clear solution was diluted using the same solvent. Finally, the fluorescence intensity of different concentrations was measured by following the aforementioned fluorescence assay procedures (as described in Sect. 2.4). The recoveries in the eye formulation were calculated by calculating the found concentration using the constructed calibration curve. Recovery is expressed as a percentage, using the formula: Recovery (%) = found concentration / taken concentration) ×100.

### Assay of BN in Artificial Aqueous Humor

The aqueous humor test solution was prepared by dissolving specific electrolytes, glucose, urea, ‎albumin, and other components in water. The ingredients were added sequentially and the pH ‎was adjusted to 7.21 using 1.0 M HCl. After filtration, the solution was stored at − 20 °C until ‎further analysis. The prepared aqueous humor was diluted 100-fold with distilled water to ‎eliminate matrix interference. ‎ For the analysis, the artificial aqueous humor was diluted 100-fold with distilled water to minimize potential matrix interferences. To perform the assay, 1.0 mL of the diluted aqueous humor was transferred into a series of 10.0 mL volumetric flasks. BN standard solutions at three different concentrations (100, 700, and 1000 ng/mL) were then added to the respective flasks and mixed thoroughly. Subsequently, 1.5 mL of 2.0% w/v SDS solution and 1.0 mL of 0.2 mol/L acetate buffer (pH 3.6) were added to each flask to enhance fluorescence intensity. The final volume was adjusted to 10.0 mL with distilled water, and the solutions were mixed well before fluorescence measurement at 446 nm. Additionally, a blank control experiment was conducted under the same conditions using artificial aqueous humor without the addition of BN to assess any background fluorescence contribution from the matrix components.

## Results and Discussion

**Fig. 1:**
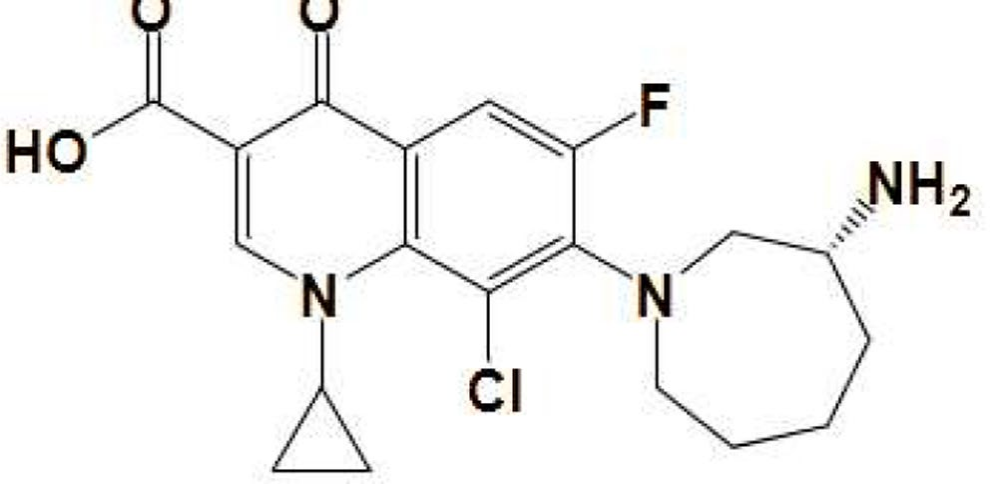
Chemical structure of BN

**Fig. 2:**
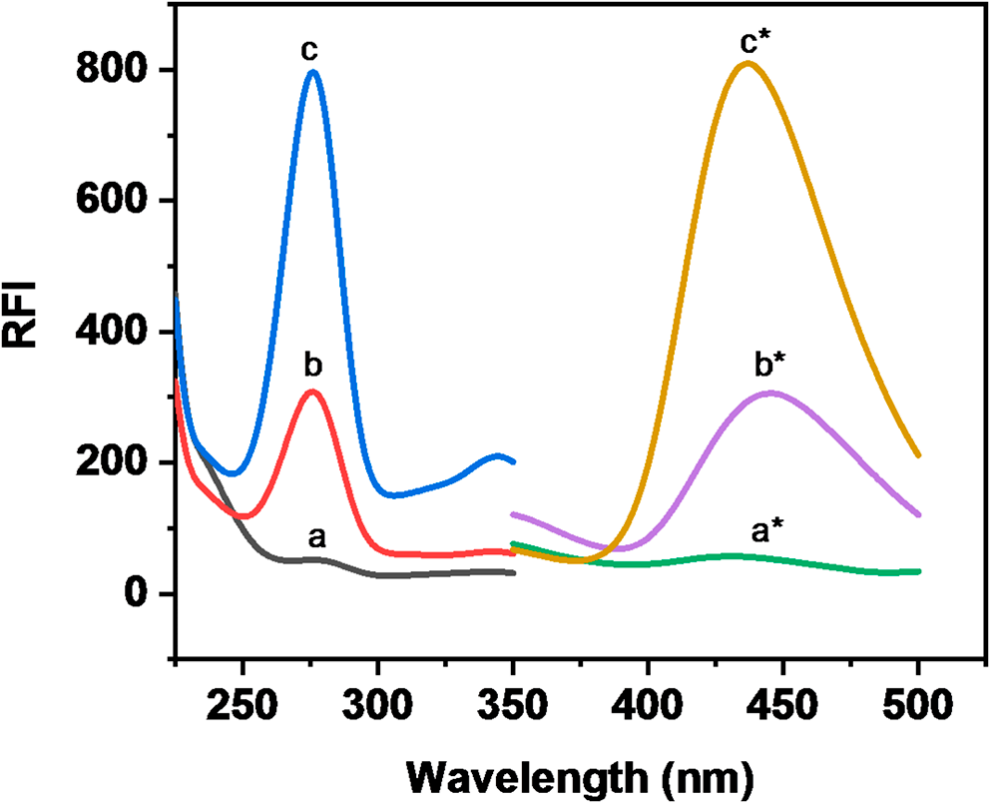
Excitation and emission spectra of BN (100 ng/mL). Where (a and a*) represent the excitation and emission spectra of SDS in acetate buffer (pH = 3.6), (b and b*) represent the excitation and emission spectra of 100 ng/ mL of BN in acetate buffer (pH = 3.6), (c and c*) represent the excitation and emission spectra of 100 ng/ mL of BN with SDS in acetate buffer (pH = 3.6)

BN (Fig. 1) contains a quinolone nucleus which is responsible for its inherent fluorescence. Notably, BN exhibits a native fluorescence at 446 nm after excitation at 274 nm (Fig. 2). We have tried to propose a new fluorimetric approach for the analysis of BN in different matrices, by boosting its emission intensity using a micellar system. As seen in Fig. 2, the fluorescence intensity of BN in the SDS micellar system has been increased (about 2.6-fold enhancement). This fluorescence enhancement is attributed to the micellar media which provide a quite different microenvironment around the studied drug than its presence in an aqueous solution without micelle formation. This different microenvironment restricts the free rotation of BN, which typically competes with luminescent emission. As a result, the likelihood of non-radiative processes is reduced, while the relatively high viscosity of the surrounding medium prevents quenching by molecular oxygen [30]. All of the above-mentioned participated in improving the intrinsic fluorescence of BN. In this way, we have achieved a great improvement in the native fluorescence emission of BN. This fluorescenceˈs amplification led to a highly sensitive fluorimetric approach for quantifying BN and achieved a remarkably low detection limit for the studied drug.‎.

### Optimization of Experimental Conditions

#### Influence of pH and Volume of Buffer

**Fig. 3:**
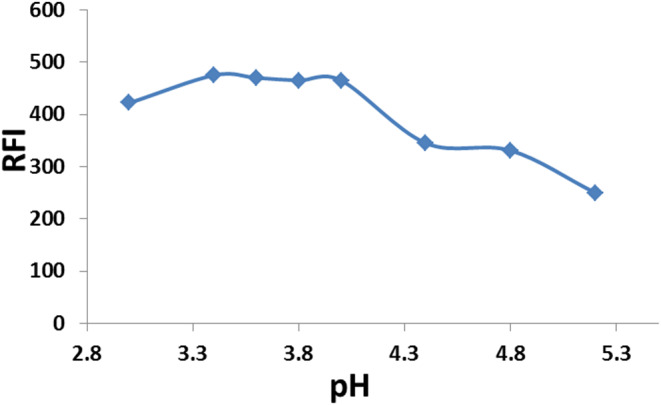
Effect of the pH on the fluorescence intensity of BN (50 ng/mL) with 2.0% w/v SDS

The effect of pH on the boosted fluorescence intensity of BN in the SDS micellar system was studied using an acetate buffer (0.2 mol/L). It was found that maximum emission was achieved at a pH of 3.6, so pH 3.6 was chosen as the optimum pH value ‎(Fig. 3). Increasing the pH value above pH 3.6 caused a gradual decrease in the emission intensity.

**Fig. 4:**
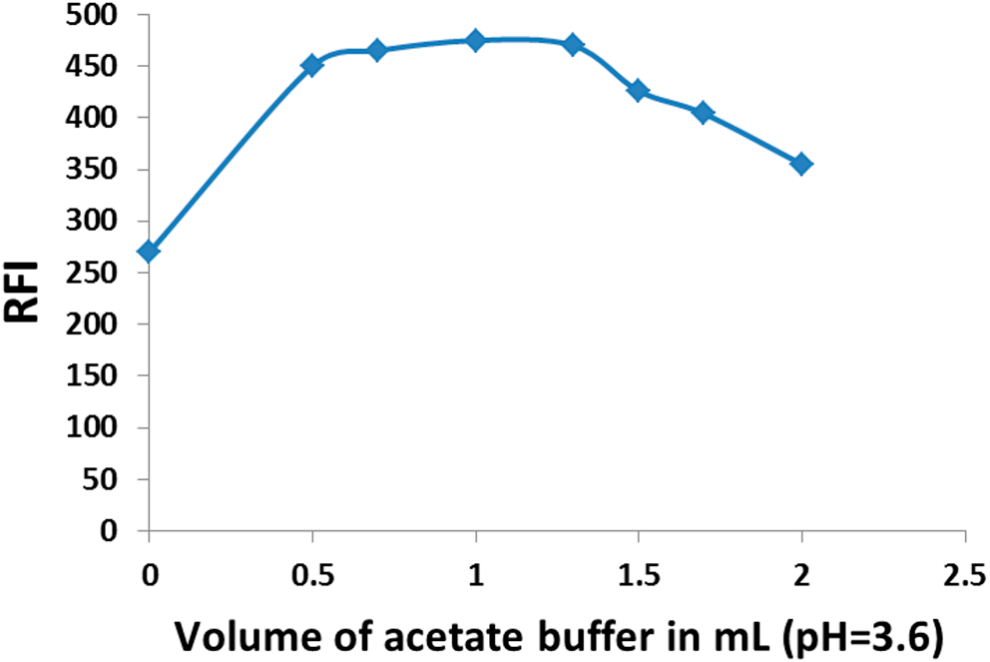
Effect of different volumes of 0.2 mol/L acetate buffer (pH = 3.6) on the fluorescence intensity of BN (50 ng/mL) with 2.0% w/v SDS

The effect of buffer volume was then examined using different volumes (0–2.0 mL) of the acetate buffer (pH = 3.6). According to the data shown in (Fig. 4), the maximum fluorescence response was attained with 1.0 mL of acetate buffer. As a result, 1.0 mL of 0.2 mol/L acetate buffer (pH = 3.6) was employed for subsequent measurements as the ideal buffer volume.

#### Effect of Different Surfactants

**Fig. 5:**
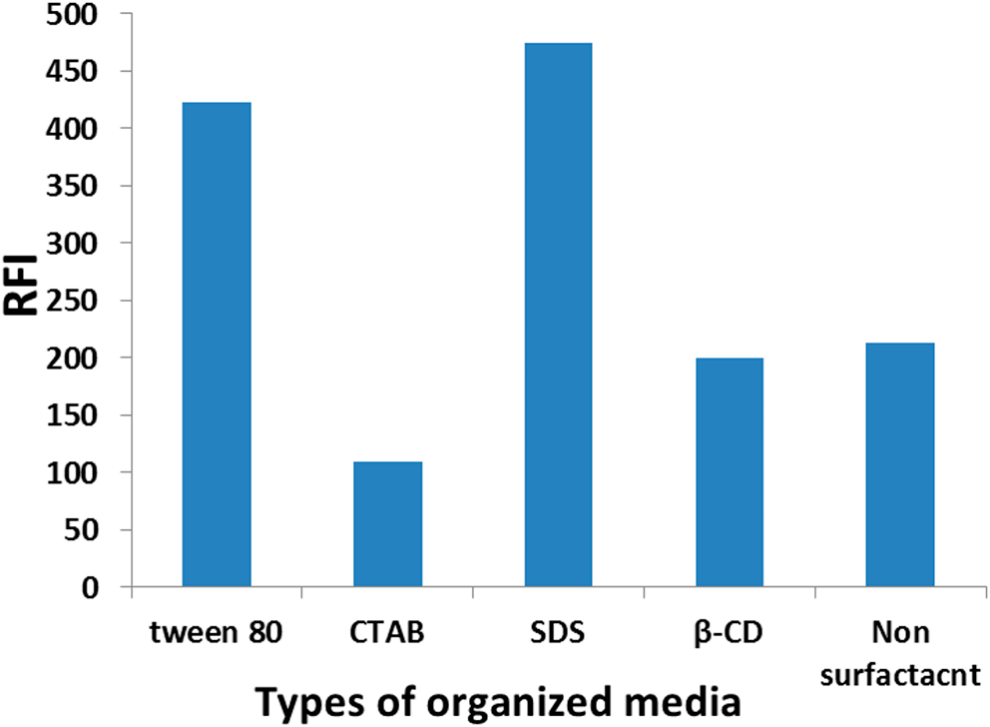
Effect of the type of organized media (β-CD, CTAB, Tween-80 and SDS) on the fluorescence intensity of BN (50 ng/ mL) at pH 3.6

BN’s fluorescence behavior in different micellar mediums was investigated with the use of non-ionic surfactants (Tween-80, β-CD), cationic surfactants (CTAB), and anionic surfactant (SDS). Interestingly, the SDS system significantly increased the emission response of the BN molecule (Fig. 5)‎.

#### Effect of SDS Volume

**Fig. 6:**
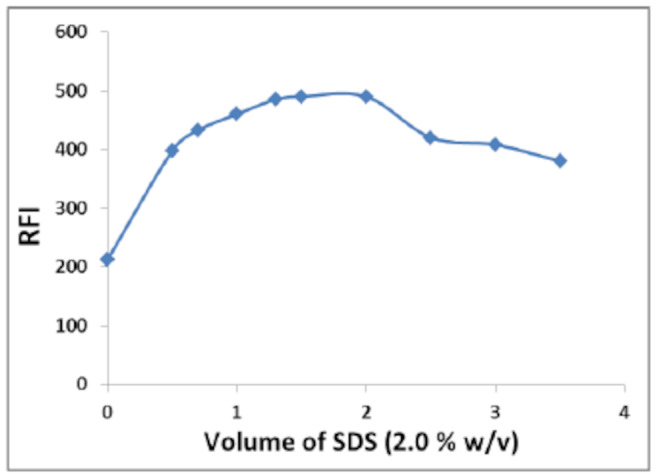
Effect of volume of 2.0% w/v SDS on the fluorescence intensity of BN (50 ng/mL) at pH 3.6

‎We also investigated how the SDS volume (2.0% w/v) affected the fluorescence intensity of BN. As represented in Fig. 6, rising SDS volume led to a commensurate rise in fluorescence intensity up to 2.0 mL of SDS solution, after which there was no noticeable rise in fluorescence intensity. Consequently, the ideal volume for a 2.0% w/v SDS solution is to be 1.5 mL and used for further measurements.

#### Effect of Diluting Solvent

BN’s fluorescence intensity in the presence of SDS was examined in relation to several dilution solvents utilizing distilled water, methanol, acetonitrile, ethanol, and acetone. Distilled water was shown to be the most effective solvent for dilution since it produced the maximum fluorescence intensity. However, the introduction of other alcohols resulted in decreased fluorescence intensity. This drop in fluorescence intensity is ‎associated with the denaturation action of short-chain alcohols on micelles [31]. Alcohols inhibit micelle formation by altering solvent characteristics and reducing micelle size, resulting in surfactant disintegration [32].

### Method Validation

The developed method was verified in terms of linearity, LOD, LOQ, precision, and accuracy in accordance with the ICH recommendations for the validation of analytical methods [33].

#### Linearity

Six standard solutions of BN, each with three replicates, were assessed for the micelle-enhanced fluorescence approach. The calibration graph was constructed by plotting fluorescence intensity against the concentrations of the BN drug (Figure S1). In the concentration range of 5.0–100 ng/mL, the relative fluorescence intensity was rectilinear (regression equation is Y = 8.02X_ng/mL_-7.19). Table 1 lists the different analytical performance parameters obtained by the developed method.

**Table 1:** Analytical performance results for the determination of BN by the developed spectrofluorimetric method

Parameter	Data for BN
λ_ex_ (nm)	274
λ_em_ (nm)	446
Linear range (ng/mL)	5.0-100
LOD (ng/mL)	0.64
LOQ (ng/mL)	1.93
Correlation coefficient (r)	0.9993
Slope (S)	8.06
Intercept	-7.19
Standard deviation of intercept (σ)	1.55
Standard deviation of slope ($\:{\mathbf{S}}_{\mathbf{s}}$)	0.042

#### Method Sensitivity

By assessing the lowest analyte concentration that can be detected and quantified, LOD and LOQ were calculated. These limits were calculated using the formula“LOD = 3.3σ/S and LOQ = 10σ/S”, where σ is the standard deviation of intercept and S is the slope of the calibration graph. LOD and LOQ were found to be 0.64 and 1.93 ng/mL, respectively (Table 1).

#### Accuracy and Precision

To assess the accuracy of our new fluorimetric approach for measuring the BN drug, we evaluated five concentrations across the developed method linear range. Our approach was shown to be highly accurate, with recoveries ranging from ‎98.15 ± 1.92‎% to ‎103.98 ± 1.01‎% (see Table 2). Furthermore, we evaluated the precision and reproducibility of our proposed approach. This involved evaluating the relative standard deviation (%RSD) at three different BN concentrations (10, 50, and 100 ng/mL). Table 3 shows low %RSD values (less than ‎2.0%). Our proposed method provides excellent precision, resulting in consistent and repeatable readings.

**Table 2:** Accuracy study for BN acheived by the developed spectrofluorimetric method

Taken concentration (ng/mL)	Found concentartion (ng/mL)	% Recovery^a^ ± SD
10	10.08	100.85 ± 1.50
30	31.19	103.98 ± 1.01
50	50.99	101.99 ± 1.56
90	92.83	103.15 ± 1.99
100	98.15	98.15 ± 1.92

^a^Mean of three determinations

**Table 3:** Intra and inter-day precision data for BN in pure state evaluated by the developed spectrofluorimetric method

Parameter	BN	
Conc. (ng/mL)	**% Recovery** ^**a**^ **± SD**	**%RSD**
Intra-day precision		
10	99.35 ± 1.18	1.18
50	102.49 ± 1.15	1.12
100	100.05 ± 1.03	1.03
Inter-day precision		
10	102.44 ± 1.08	1.05
50	99.59 ± 1.08	1.08
100	99.36 ± 0.67	0.67

^a^Mean of three determinations

### Application to Pharmaceutical Eye Drops

The BN drug in eye drops was analyzed using the reported spectrophotometric method [14] by measuring the absorbance of the drug at λ_max_ 289 nm in distilled water using a UV/Vis double-beam spectrophotometer. Both the developed and reported methods’ outcomes were statistically compared. There were no significant differences between the calculated and theoretical values when comparing the results of the suggested and reported procedures using the student’s t and F-tests (Table 4). This demonstrated the suggested method’s high level of accuracy and precision.

**Table 4:** Determination of the studied BN drug in eye drops using the proposed method and reporting methods

Dosage form	Labeled content	% Recovery ^a^ ± SD		t- value^b^	F- value^b^
		Proposed method	Reference method [14]		
**Pixivance** ^**®**^ **Eye drop**	6.0 mg/mL	100.77 ± 1.61	99.34 ± 0.92	1.71	3.03

^a^Mean of three determinations

^b^Theoretical values at 95% confidence limit: *t* = 2.31, *F* = 6.39

### Application of BN in Artificial Aqueous Humor

BN in artificial aqueous humor was measured using our highly sensitive method. The process was followed after adding different concentrations of BN to aqueous humor samples. High recoveries were achieved from 98.82 ± 1.89% to 103.08 ± 1.60%. The effectiveness of this technique for assessing BN levels in spiked aqueous humor samples is shown by the high recovery rates in Table 5.

**Table 5:** Recovery study of aqueous humor samples spiked with BN drug

Spiked Concentration (ng/mL)	Found Concentration (ng/mL)	Recovery^a^ (%) ± SD
**10**	10.30	103.08 ± 1.60
**70**	69.17	98.82 ± 1.89
**100**	101.22	101.22 ± 1.25

^a^Average of three determinations

## Conclusion

This work introduces a micelle-enhanced spectrofluorimetric approach for the quantification of BN in ophthalmic preparations and spiked aqueous humor. By employing SDS micelles, the fluorescence intensity of BN was significantly enhanced by 2.6-fold, enhancing both sensitivity and selectivity. The optimized method displayed a wide linear range (5.0–100 ng/mL), and a low LOD of 0.64 ng/mL, making it highly appropriate for pharmaceutical drug analysis. Compared to reported chromatographic and electrochemical methods, this approach provides ‎ a simple, cost-effective, and highly sensitive alternative method of analysis for BN without the need for complex derivatization or expensive instrumentation. Overall, this work offers a new and practical method for the routine assay of BN in ophthalmic preparations, with potential implementations in clinical quality control and pharmaceutical industries.

## Electronic Supplementary Material

Below is the link to the electronic supplementary material.

Supplementary Material 1

## Data Availability

No datasets were generated or analysed during the current study.
