# Supplementary material for: Exploring the Role of Sodium Dodecyl Sulfate Surfactant in Enhancing the Fluorescence Properties of Besifloxacin Fluoroquinolone; Application to Eye Formulations and Artificial Aqueous Humor
Source: J Fluoresc. 2025 May 9;35(11):10783–9. doi: 10.1007/s10895-025-04310-1 (PMC12718253; doi:10.1007/s10895-025-04310-1)
Supplement: Supplementary file 1 — Supplementary Material 1 [file 10895_2025_4310_MOESM1_ESM.docx]

**Supplemetray Data**

**Exploring the Role of Sodium Dodecyl Sulfate Surfactant in Enhancing the Fluorescence Properties of Besifloxacin** **Fluoroquinolone; Application to Eye Formulations and Artificial Aqueous Humor.**

Islam M. Mostafa^a,b*^, Demiana W. Fakhry ^c^, Mohamed A. Abdelshakour ^c^, Deena A. M. Nour El-Deen^a,b^

^a^ Analytical Chemistry Department, Faculty of Pharmacy, Minia University, Minia, 61511, Egypt

^b^ Analytical Chemistry Department, Faculty of Pharmacy, Minia National University, New Minia, 61511, Egypt

^c^ Department of Pharmaceutical Analytical Chemistry, Faculty of Pharmacy, Sohag University, Sohag 82524, Egypt.

^
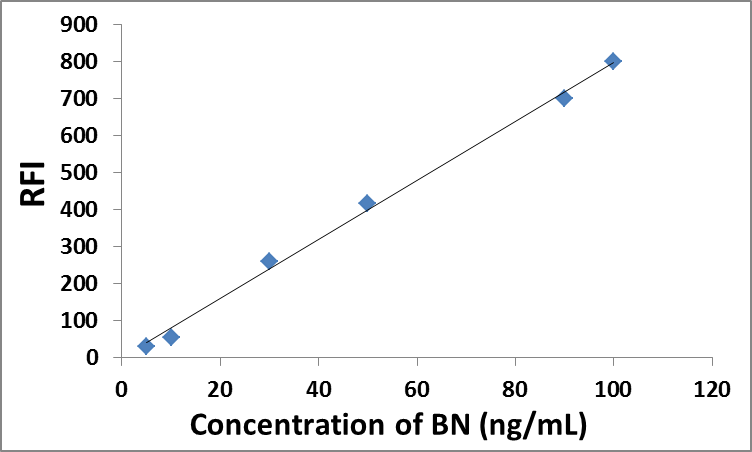
^

**Figure S1**. Calibration graph for BN constructed by the developed method.
